# Comparison of Properties among Dendritic and Hyperbranched Poly(ether ether ketone)s and Linear Poly(ether ketone)s

**DOI:** 10.3390/molecules21020219

**Published:** 2016-02-16

**Authors:** Atsushi Morikawa

**Affiliations:** Department Biomolecular Functional Engineering, Ibaraki University, 4-12-1, Nakanarusawa, Hitachi, Ibaraki 316-8511, Japan; atsushi.morikawa.reg@vc.ibaraki.ac.jp; Tel.: +81-294-38-5070; Fax: +81-294-38-5078

**Keywords:** poly(ether ether ketone) dendrimers, hyperbranched polymers, linear poly(ether ketone)s, solubility, reduced viscosities, thermal properties, molecular size

## Abstract

Poly(ether ether ketone) dendrimers and hyperbranched polymers were prepared from 3,5-dimethoxy-4′-(4-fluorobenzoyl)diphenyl ether and 3,5-dihydroxy-4′-(4-fluorobenzoyl)diphenyl ether through aromatic nucleophilic substitution reactions. 1-(*tert*-Butyldimethylsiloxy)-3,5-bis(4-fluorobenzoyl)benzene was polycondensed with bisphenols, followed by cleavage of the protective group to form linear poly(ether ketone)s having the same hydroxyl groups in the side chains as the chain ends of the dendrimer and hyperbranched polymers. Their properties, such as solubilities, reduced viscosities, and thermal properties, were compared with one another. Similar comparisons were also carried out among the corresponding methoxy group polymers, and the size of the molecules was shown to affect the properties.

## 1. Introduction

Dendritic macromolecules with branching structures are classified as dendrimers or hyperbranched polymers. Dendrimers are well-defined macromolecules consisting of dendritic units and terminal units that exhibit precise tree-like structures [1] and constructed via step-by-step sequences, requiring isolation and purification [2,3,4,5,6,7,8,9,10,11,12]. Hyperbranched polymers are prepared by one-step polymerization of AB_x_-type monomers, resulting in polydispersed macromolecules consisting of dendritic units, linear units, and terminal units that exhibit irregular structures [13,14,15,16,17,18,19,20,21,22,23,24,25,26,27,28,29,30,31,32]. However, the properties of hyperbranched polymers are similar to those of dendrimers [15,16]. A key characteristic of these polymers is a branched structure with a large number of chain end groups, to which their low ability of forming intermolecular entanglements is generally ascribed [16]. Hyperbranched polymers and dendrimers have been modified by introduction of functional groups to the chain-end or chain backbone, and a variety of applications, such as encapsulators [33,34], composite materials [35,36,37,38], ion-exchange membranes [39], gas separation membrane [40], photoresistors [41], nonlinear optical devices [42,43], diodes [44], cross-linkers [45,46,47] and heterogeneous catalyst [48,49,50,51] have been investigated. In addition, the applications to size-sensitive host [52], subnanomaterial synthesis [53], pH-responsive agents [54], MRI agents [55,56,57] and drug delivery [58] have been examined due to the precise nanostructures found in dendrimers. The feasibility of these applications was thought to benefit from the presence of a large number of functional sites within the compact space. Linear polymers having functional sites in the side chains are structurally similar, thus the same applications may be possible by using linear polymers having functional sites in the side chains. Therefore, it is important to compare the properties of dendrimers and hyperbranched polymers with those of linear polymers having the same functional groups in the side chains as the end groups of dendrimers and hyperbranched polymers in order to examine the functionality derived from the size of the molecules. However, such a comparison has only been reported by Wooly and coworkers in polyesters [15]. The glass transition temperature *T*_g_s of the dendrimer, the hyperbranched polyester, and the linear polyester were similar, but the dendrimer and the hyperbranched polyester showed higher solubility and special chemical reactivity.

In this study, linear poly(ether ketone)s having hydroxyl groups in the side chain were prepared using hydroxyl-protected 3,5-bis(4-fluorobenzoyl)phenol [14]. The properties of the poly(ether ether ketone) dendrimers, poly(ether ether ketone) hyperbranched polymers, and the linear poly(ether ketone)s, such as solubilities, reduced viscosities, and thermal properties, were compared. The properties of the corresponding polymers having methoxy groups were also compared to evaluate the effect of the terminal group.

## 2. Results and Discussion

### 2.1. Poly(ether ether ketone)s Dendrimers

**Scheme 1:**
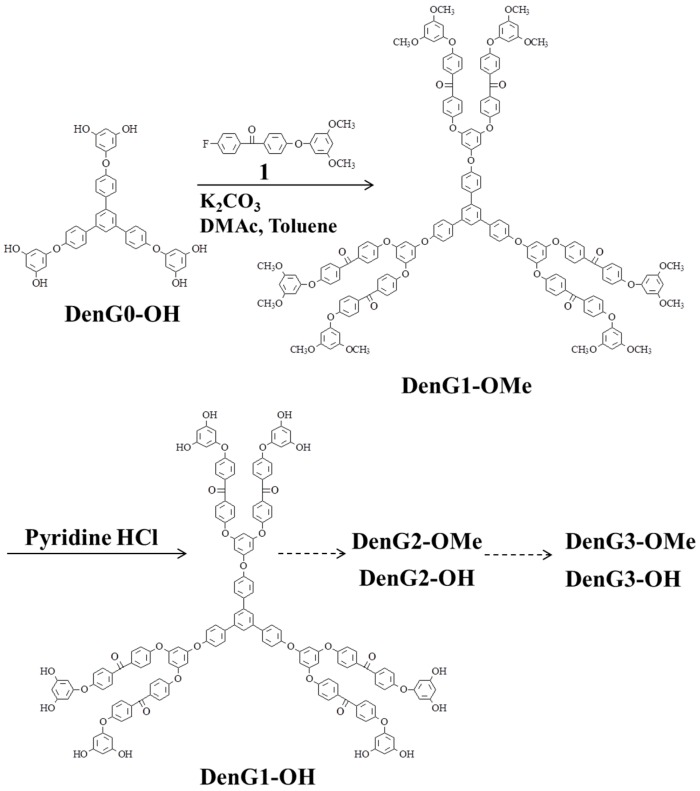
Synthesis of poly(ether ether ketone) dendrimers.

Poly(ether ether ketone) dendrimers were synthesized using 3,5-dimethoxy-4′-(4-fluorobenzoyl)-diphenyl ether (**1**) and 1,3,5-tris[*p*-(3,5-dihydroxyphenoxy)phenyl]benzene (**DenG0-OH**) as the building block and starting core, respectively, as shown in Scheme 1 [16].

Reaction of **DenG0-OH** with **1** in the presence of potassium carbonate in *N*,*N*-dimethyl-acetamide (DMAc) and toluene gave the first generation dendrimer (**DenG1-OMe**) [59]. The water formed during the reaction was removed as an azeotrope. The methoxy groups of **DenG1-OMe** were converted to hydroxyl terminal first generation (**DenG1-OH**) by the treatment with pyridine hydrochloride at 240 °C. Further reaction of **DenG1-OH** with **1** gave the second generation dendrimer (**DenG2-OMe**), and **DenG2-OMe** was converted to hydroxyl terminal second generation (**DenG2-OH**). One more repetition of the procedure gave the third generation dendrimer (**DenG3-OMe**), and hydroxyl terminal third generation (**DenG3-OH**). All the dendrimers were purified by silica gel column chromatography, and the final isolated yields of **DenG1-OMe**, **DenG2-OMe**, and **DenG3-OMe** were 92%, 83%, and 64%, respectively, and those of **DenG1-OH**, **DenG2-OH**, and **DenG3-OH** were 95%, 91%, and 87%, respectively. The growth and purity were confirmed by gel permeation chromatography (GPC) and MALDI-TOF. 

### 2.2. Hyperbranched Poly(ether ether ketone)s

**Scheme 2:**
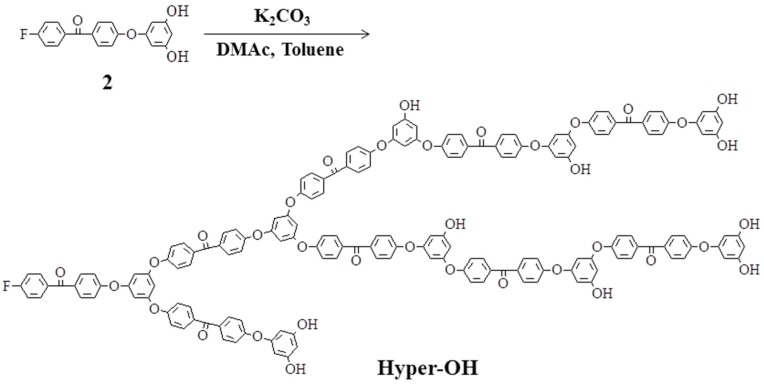
Synthesis of hyperbranched poly(ether ether ketone).

**Figure 1:**
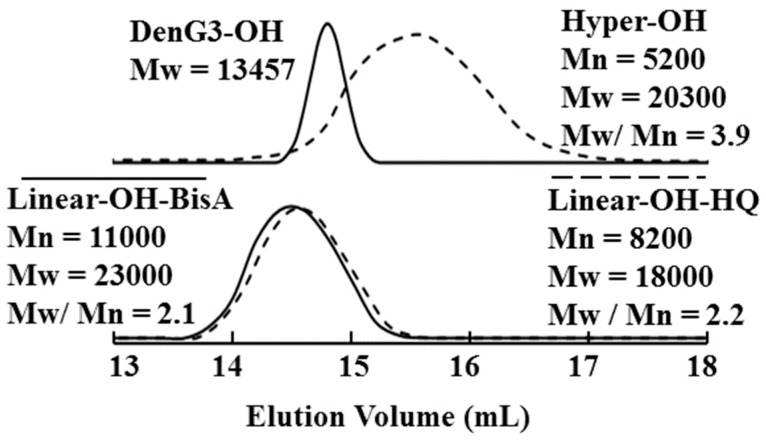
Gel permeation chromatography of **DenG3-OH**, **Hyper-OH**, **linear-OH-BisA**, and **linear-OH-HQ**.

Hyperbranched poly(ether ether ketone)s (**Hyper-OH**) were synthesized by one-step polycondensation of AB_2_-type monomer, 3,5-dihydroxy-4′-(4-fluorobenzoyl)diphenylether (**2**) (Scheme 2) [31]. The polymerization proceeded in the presence of K_2_CO_3_ at 120–165 °C using a mixture of DMAc and toluene as the solvent [59]. The water formed during the reaction was removed as an azeotrope to promote polymerization. The yield was 88%, and GPC analysis of **Hyper-OH** gave a number-average molecular weight (Mn) of 5200 and a weight-average molecular weight (Mw) of 20300, which were calibrated against polystyrene standards (Figure 1). With the help of the dendrimers and model compound, ^1^H-NMR studies revealed that the degree of branching of **Hyper-OH** was 52%. Hyperbranched poly(ether ether ketone)s (**Hyper-OMe**) having methoxy groups in the chain ends were prepared by reaction of **Hyper-OH** with dimethyl sulfate.

### 2.3. Linear Poly(ether ketone)s Having Hydroxyl Groups in the Side Chains

Linear poly(ether ketone)s having hydroxyl groups in the side chains can be synthesized by polycondensation of an AB-type monomer derived by protecting only one hydroxyl group in **2**, followed by cleavage of the protective group. Since we could not synthesized one such hydroxyl group protected compound, 3,5-bis(4-fluorobenzoyl)phenol (**3**) was used for the synthesis of the linear type polymer, which was similar to the linear type poly(ether ether ketone) from **2**.

**Scheme 3:**
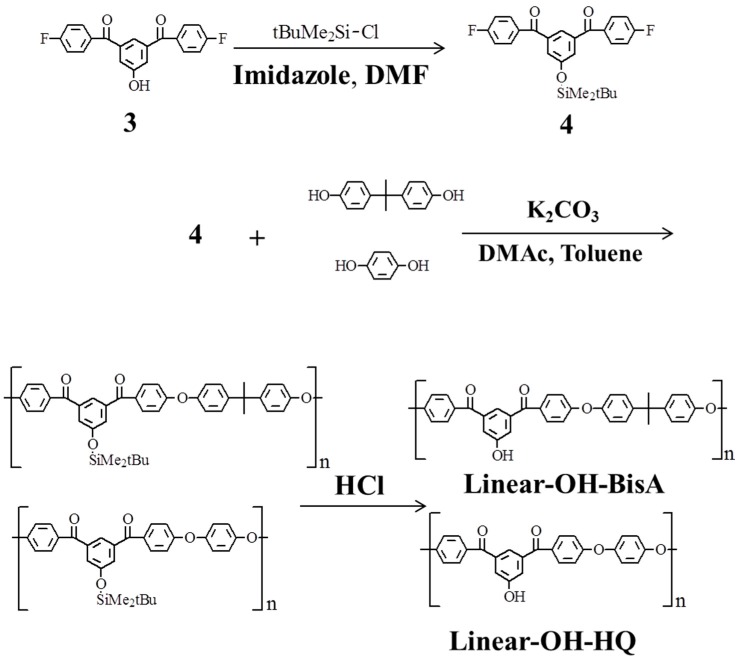
Synthesis of linear poly(ether ketone)s having hydroxyl groups in the side chains.

1-(*tert*-Butyldimethylsilyloxy)-3,5-bis(4-fluorobenzoyl)benzene (**4**), derived from **3** [60,61], was polycondensed with bisphenols, bisphenol A and hydroquinone, and followed by cleavage of the protective group to form linear poly(ether ketone)s (Scheme 3). Polymerization of **4** with the bisphenols was carried out in the presence of K_2_CO_3_ at 120–165 °C using a mixture of DMAc and toluene as the solvent [59]. The water formed during the reaction was removed as an azeotrope to promote the polymerization. The polymerizations proceeded homogeneously without gelation. The mixture was poured into water and treated with hydrochloric acid [60,61] to form hydroxyl linear poly(ether ketone)s (**Linear-OH-BisA** and **Linear-OH-HQ**) having hydroxyl groups in the side chains. The formations of **Linear-OHs** were confirmed by appearance of adsorption bands at around 1150 cm^−1^ characteristic due to ether group and 3600–3200 cm^−1^ due to hydroxyl group in the IR spectra.

**Figure 2:**
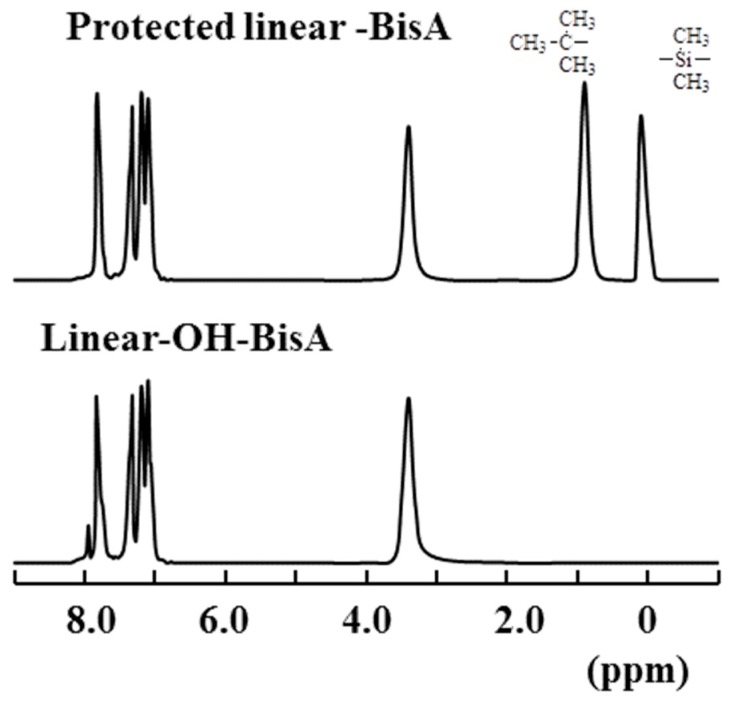
^1^H-NMR spectra of protected **Linear-OH-BisA** and **Linear-OH-BisA**.

In the ^1^H-NMR spectra (Figure 2) measured for **Linear-OH-BisA**, the signals assigned to the *tert*-butyldimethylsilyl group were not observed after treatment with hydrochloric acid, and the deprotection reaction proceeded completely. We also attempted to synthesized 3,5-bis(4-fluoro-benzoyl)anisole (**5**), the methyl protected compound of **3**, for linear poly(ether ketone)s as in the case of the dendrimers. However, while poly(ether ketone) dendrons could be synthesized from **5** [3], the methyl groups could not be completely converted to hydroxyl groups in this synthesis of **Linear-OH-BisA** and **Linear-OH-HQ**. The yields of **Linear-OH-BisA** and **Linear-OH-HQ** were 87% and 85%, respectively. GPC analysis of **Linear-OH-BisA** gave Mn of 11000 and Mw of 23000, and that of **Linear-OH-HQ** gave Mn of 8200 and Mw of 18000 (Figure 1). Their molecular weight distributions were about 2.0, and smaller than that of **Hyper-OH**. Molecular weight distribution of Hyperbranched polymer is thought to be higher than that linear polymer due to distribution of DB as well as molecular weight. Linear poly(ether ketone)s (**Linear-OMe-BisA** and **Linear-OMe-HQ**) having methoxyl groups in the side chains were prepared by polycondensation of **5** and the bisphenols.

### 2.4. Comparison of Properties

The solubilities, solution viscosities, and thermal properties of poly(ether ether ketone) dendrimers, hyperbranched poly(ether ether ketone)s, and linear poly(ether ketone)s having hydroxyl groups in the side chains were compared, and the effect of the size of the molecules on the properties was evaluated. Those of **DenG3-OMe**, **Hyper-OMe**, **Linear-OMe-BisA**, and **Linear-OMe-HQ** were also compared to evaluate effect of terminal group.

Table 1 shows a solubility comparison between **DenG3-OMe**, **Hyper-OMe**, **Linear-OMe-BisA** and **Linear-OMe-HQ**. They were all soluble in NMP, DMAc, and tetrahydrofuran at room temperature. **DenG3-OMe** and **Hyper-OMe** were also soluble in pyridine and non-polar solvents, such as chloroform and toluene, showed broad solubility, but **Linear-OMe-BisA** and **Linear-OMe-HQ** were insoluble in pyridine and toluene. Table 2 shows solubilities of **DenG3-OH**, **Hyper-OH**, **Linear-OH-BisA** and **Linear-OH-HQ**. They were soluble in pyridine as well as NMP, DMAc, and tetrahydrofuran, and the functional groups in the termini or side chains were reflected in the solubility.

The effect of the size of the molecules on the solubility was observed in alkaline aqueous solution. **DenG3-OH** was soluble in 1 M aqueous NaOH solution, **Hyper-OH** was soluble upon heating, and **Linear-OH-BisA** and **Linear-OH-HQ** were insoluble even with heating. **DenG3-OH** was also soluble in methanol. 

**Table 1:** Solubilities of **DenG3-OMe**, **Hyper-OMe**, **Linear-OMe-BisA**, and **Linear-OMe-HQ**.

Methoxy Group Polymer	NMP	DMAc	CHCl_3_	Pyridine	THF	Toluene	CH_3_OH	NaOH Aquation
**DenG3-OMe**	++	++	++	++	++	++	--	--
**Hyper-OMe**	++	++	++	++	++	++	--	--
**Linear-OMe-BisA**	++	++	++	--	++	--	--	--
**Linear-OMe-HQ**	++	++	--	--	++	--	--	--

Solubility: ++, soluble at room temperature; +, soluble on heating, --, insoluble. Abbreviation of solvent: NMP, *N*-methyl-2-pyrrolidinone; DMAc, *N*,*N*-dimethylacetamide; THF, tetrahydrofuran.

**Table 2:** Solubilities of **DenG3-OH**, **Hyper-OH**, **Linear-OH-BisA**, and **Linear-OH-HQ**.

Hydroxy Group Polymer	NMP	DMAc	CHCl_3_	Pyridine	THF	Toluene	CH_3_OH	NaOH Aquation
**DenG3-OH**	++	++	--	++	++	--	++	++
**Hyper-OH**	++	++	--	++	++	--	--	+
**Linear-OH-BisA**	++	++	--	++	++	--	--	--
**Linear-OH-HQ**	++	++	--	++	++	--	--	--

Solubility: ++, soluble at room temperature; +, soluble on heating, --, insoluble. Abbreviation of solvent: NMP, *N*-methyl-2-pyrrolidinone; DMAc, *N*,*N*-dimethylacetamide; THF, tetrahydrofuran.

**Figure 3:**
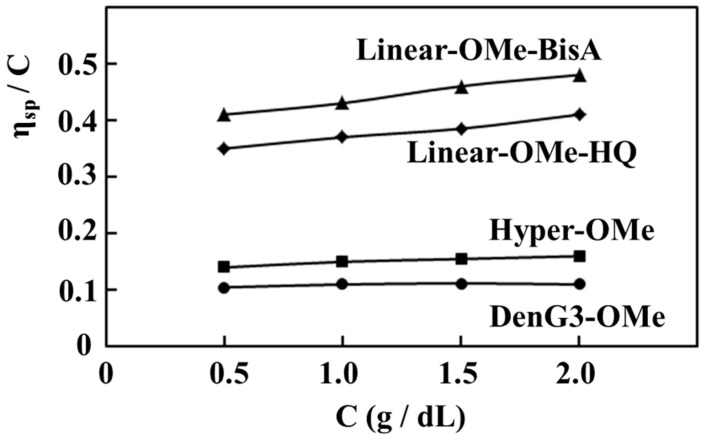
Reduced viscosities of **DenG3-OMe**, **Hyper-OMe**, **Linear-OMe-BisA**, and **Linear-OMe-HQ**.

Figure 3 shows the relationship between the reduced viscosities of **DenG3-OMe**, **Hyper-OMe**, **Linear-OMe-BisA**, and **Linear-OMe-HQ** and the concentration in DMAc. The reduced viscosities of **DenG3-OMe**, **Hyper-OMe**, **Linear-OMe-BisA**, and **Linear-OMe-HQ** at the concentration of 1.0 g/dL were 0.11, 0.15, 0.43, and 0.37, respectively. The reduced viscosities of **DenG3-OMe** and **Hyper-OMe** were independent of the concentration, and those of **Linear-OMe-BisA** and **Linear-OMe-HQ** increased slightly with concentration. 

**Figure 4:**
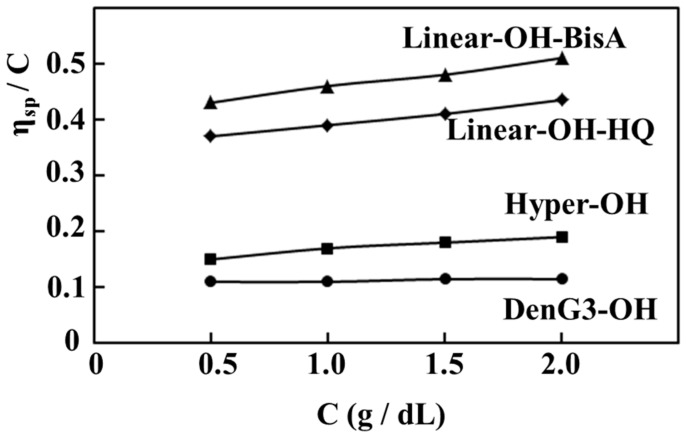
Reduced viscosities of **DenG3-OH**, **Hyper-OH**, **Linear-OH-BisA** and **Linear-OH-HQ**.

Figure 4 shows the relationship between the reduced viscosities of **DenG3-OH**, **Hyper-OH**, **Linear-OH-bisA** and **Linear-OH-HQ** and the concentration in DMAc. The reduced viscosities of **DenG3-OH**, **Hyper-OH**, **Linear-OH-BisA**, and **Linear-OH-HQ** at the concentration of 1.0 g/dL were 0.11, 0.17, 0.46, and 0.39, respectively, and the GPC results (Figure 1) are reflected in the viscosities. Their higher values suggested a higher degree of interaction with the solvent due to the polarity of the hydroxyl groups. The reduced viscosity of **DenG3-OH** was independent of the concentration, that of **Hyper-OH** increased slightly with concentration, and those of **Linear-OH-BisA** and **Linear-OH-HQ** increased more significantly than **Hyper-OH**. The reduced viscosity of the **Linear-OH**s also increased more than **Linear-OMe**s. The concentration dependence of the reduced viscosity of **Hyper-OH** slightly reflected the aspect of linear polymer. The length between branching points of **Hyper-OH** is long due to the benzophenone unit, and this effect may be observed.

**Figure 5:**
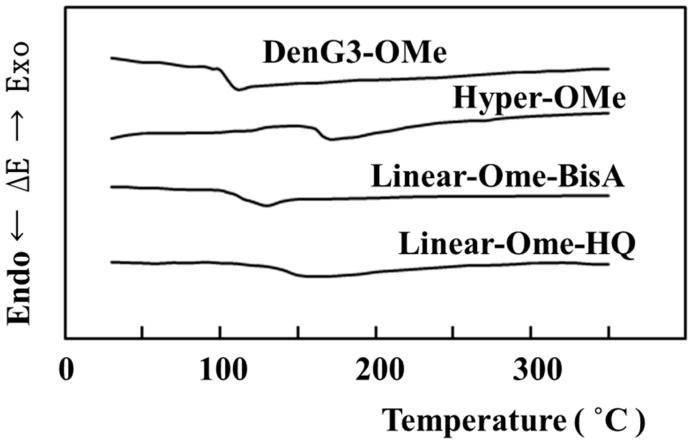
DSC curves of **DenG3-OMe**, **Hyper-OMe**, **Linear-OMe-BisA**, and **Linear-OMe-HQ**.

Thermal properties were evaluated by differential scanning calorimetry (DSC). Figure 5 shows DSC behaviors of **DenG3-OMe, Hyper-OMe**, **Linear-OMe-BisA**, and **Linear-OMe-HQ**. They seemed to be non-crystalline, and only *T*_g_ was observed. *T*_g_s of **DenG3-OMe**, **Hyper-OMe**, **Linear-OMe-BisA**, and **Linear-OMe-HQ** were 101, 160, 105 and 144 °C, respectively, and the order of increasing *T*_g_ values was **DenG3-OMe** < **Linear-OMe-BisA** < **Linear-OMe-HQ** < **Hyper-OMe**. The structure of the dendrimer molecule was near spherical due to perfect branching and compact structures, but that of the hyperbranched molecule was not near spherical due to moderate DB (52%), and the surface area of the hyperbranched molecule was larger. The interaction between the hyperbranched molecules was thought to be larger due to the larger surface area. *T*_g_ of hyperbranched molecules is thought to be higher than linear polymers due to both branching and the interaction among molecules.

**Figure 6:**
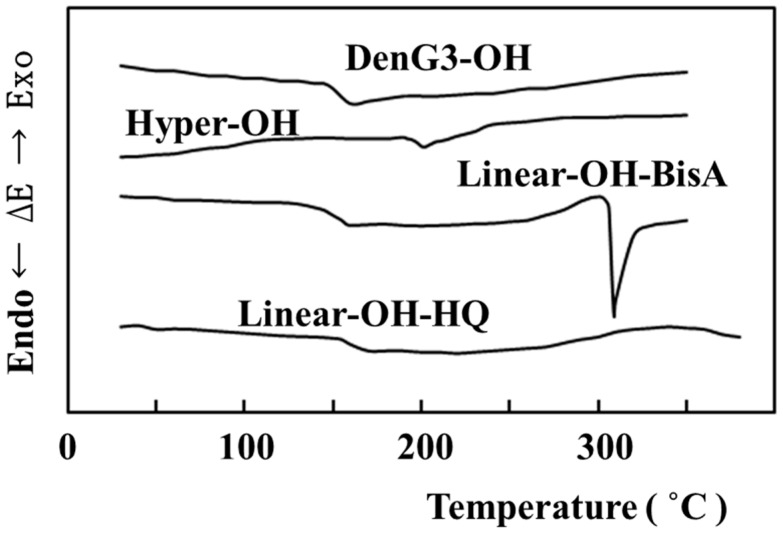
DSC curves of **DenG3-OH**, **Hyper-OH**, **Linear-OH-BisA**, and **Linear-OH-HQ**.

Figure 6 shows DSC behaviors of **DenG3-OH**, **Hyper-OH**, **Linear-OH-BisA**, and **Linear-OH-HQ**. **DenG3-OH** and **Hyper-OH** also seemed to be non-crystalline, and *T*_g_s were observed at 144 and 190 °C, respectively. **Linear-OH-BisA** exhibited *T*_g_ at 145 °C followed by exothermic crystallization at 250–305 °C, and **Linear-OH-HQ** exhibited *T*_g_ at 155 °C followed by exothermic crystallization at 280–340 °C. **Linear-OH-BisA** exhibited an endothermic melting peak at 305–320 °C. *T*_g_s of **Hyper-OMe** and **Hyper-OH** were also much higher than the corresponding dendrimers and linear polymers, and in contrast to the polyesters having hydroxyl groups [15]. *T*_g_s of hyperbranched and dendritic polyesters having hydroxyl groups in the chain ends were almost the same as the *T*_g_ of the linear polyester having hydroxyl groups in the side chains. **DenG3-OH** and **Hyper-OH** did not crystallize, but **Linear-OH-BisA** and **Linear-OH-HQ** crystallized. Dendritic polymers could not be crystallized due to their branching structures, and attachment of crystalline molecules to the chain ends as a molecular scaffold was reported to be necessary for the crystallization of hyperbranched polymers [18,24]. Since the chains of **Linear-OH-BisA** and **Linear-OH-HQ** without branching were flexible, the crystallizations were thought to be induced due to hydrogen bonding by the hydroxyl groups.

## 3. Experimental Section 

### 3.1. General Information

^1^H- and ^13^C- NMR spectra were recorded on a JNM-GSX400 FT-NMR spectrometer (JEOL, Akishima, Japan) and IR spectra were recorded on a IR 435 spectrophotometer (Shimadzu, Kyoto, Japan). GPC was carried out on a PL gel 5μ MIXED-C analytical column (Polymer Laboratories, Tokyo, Japan) with tetrahydrofuran as the eluent. Reduced viscosity was measured at various concentrations using an Ubbelohde-type capillary viscometer (Shibata, Sohka, Japan) in DMAc at 30 °C. MALDI-TOF MS were measured on a Shimadzu/Kratos Kompact MALDI II (Shimadzu) equipped with a 337 nm nitrogen laser. DSC was performed with a Shimadzu DSC-60 (Shimadzu), instrument and measurements were made at a heating rate of 10 °C min^−1^ in nitrogen. The results from first scan were displayed in Figure 5 and Figure 6.

### 3.2. Conventional Synthesis

#### 3.2.1. Third Generation Dendrimer (**DenG3-OMe**) 

In a flask, a mixture of **1** (7.05 g, 20 mmol), **DenG0-OH** (1.70 g , 2.5 mmol), potassium carbonate (2.76 g, 20 mmol), toluene (30 mL), and DMAc (60 mL) was stirred at 130 °C for 1 h. The temperature was raised to 160 °C and water formed during the reaction was removed as an azeotrope with toluene. The reaction mixture was stirred at this temperature for 1.5 h. After the reaction was complete, the mixture was cooled to about 80 °C, and the solvent was evaporated under reduced pressure of 15–20 torr. The residue was washed with water (300 mL) and extracted twice with methylene chloride (200 mL). After the combined extract was dried over anhydrous magnesium sulfate, the solvent was evaporated. Pure **DenG1-OMe** was obtained by silica gel column chromatography beginning with methylene chloride as the eluent and gradually changing to methylene chloride and ethyl acetate (20:1), yield: 92%.

**DenG1-OMe** (5.35 g, 2 mmol) was heated together with pyridine hydrochloride (50 g) at reflux temperature for about 30 min. After the reaction mixture was homogeneous, it was poured into water (1000 mL), and extracted twice with ethyl acetate (100 mL). The combined extract was dried over anhydrous magnesium sulfate. After evaporation of the solvent, pure **DenG1-OH** was obtained by silica gel column chromatography by methylene chloride and ethyl acetate (1:1). Yield: 95%.

**DenG2-OMe** was prepared by the same procedure as that for synthesis of **DenG1-OMe** using **DenG1-OH** (2.50 g, 1 mmol), 1 (6.34 g, 18 mmol), potassium carbonate (2.49 g, 18 mmol), toluene (23 mL), and DMAc (60 mL). Pure **DenG2-OMe** was obtained by silica gel column chromatography beginning with methylene chloride and ethyl acetate (25:1) as the eluent and gradually changing to methylene chloride and ethyl acetate (15:1), yield: 83%.

**DenG2-OH** was prepared by the same procedure as that for synthesis of **DenG1-OH** using **DenG2-OMe** (4.54 g, 0.7 mmol), and pyridine hydrochloride (60 g). Pure **DenG2-OH** was obtained by silica gel column by methylene chloride and ethyl acetate (3:7), yield: 91%.

**DenG3-OMe** was prepared by the same procedure as that for synthesis of **DenG1-OMe** using **DenG2-OH** (1.85 g, 0.3 mmol), **1** (3.81 g, 10.8 mmol), potassium carbonate (1.49 g, 10.8 mmol), 15 toluene (15 mL), and DMAc (30 mL). Pure **DenG3-OMe** was obtained by silica gel column chromatography beginning with methylene chloride and ethyl acetate (20:1) as the eluent and gradually changing to methylene chloride and ethyl acetate (13:1). Yield 62%; white powder; Elemental Analysis: C_888_H_630_O_117_; Calculated %C = 75.47, %H = 4.49. Observed. %C = 75.35, %H = 4.41. FT-IR/ATR ν (cm^−1^): 2940, 1655, 1590, 1225, and 1160 cm^−1^; TOF-MS: 14133, C_888_H_630_O_177_ (Mw = 14131); ^1^H-NMR (CDCl_3_) ppm: 7.83–7.47 (m,168H, Ar), 7.68 (s, 3H, Ar), 7.65 (d, 6H, *J* = 8.8 Hz, Ar), 7.15 (d, 6H, *J* = 8.8 Hz, Ar), 7.10–7.06 (m,120H, Ar), 7.04 (d, 48H, *J* = 8.8 Hz, Ar), 6.58 (m, 4H, Ar), 6.48 (t, 3H, *J* = 2.2 Hz, Ar), 6.28 (t, 24H, *J* = 2.2 Hz, Ar), 6.22 (d, 48H, *J* = 2.2 Hz, Ar), 3.75 (s, 144H, -CH_3_); ^13^C-NMR (CDCl_3_) ppm: 193.9 (C=O), 193.9 (C=O), 193.8 (C=O), 161.9 (Ar), 161.1 (Ar), 160.1 (Ar), 160.1 (Ar), 160.0 (Ar), 159.9 (Ar), 159.9 (Ar), 159.8 (Ar), 158.5 (Ar), 158.5 (Ar), 158.5 (Ar), 158.2 (Ar), 157.5 (Ar), 155.6 (Ar), 141.6 (Ar), 137.2 (Ar), 133.5 (Ar), 133.3 (Ar), 133.2 (Ar), 133.2 (Ar), 133.0 (Ar), 132.3 (Ar), 132.2 (Ar), 132.2 (Ar), 132.1 (Ar), 131.9 (Ar), 128.9 (Ar), 124.7 (Ar), 120.0 (Ar), 118.5 (Ar), 118.2 (Ar), 118.1 (Ar), 117.6 (Ar), 106.3 (Ar), 106.3 (Ar), 106.2 (Ar), 105.5 (Ar), 105.3 (Ar), 98.6 (Ar), 96.8 (Ar), 55.5(-CH_3_).

#### 3.2.2. Hydroxyl Terminal Third Generation (**DenG3-OH**)

**DenG3-OH** was prepared by the same procedure as that used for synthesis of **DenG1-OH** using **DenG3-OMe** (2.11 g, 0.15 mmol), and pyridine hydrochloride (80 g). Pure **DenG3-OH** was obtained by silica gel column by methylene chloride and ethyl acetate (3:7). Yield 87%; white powder; Elemental Analysis: C_840_H_534_O_117_; Calculated %C = 74.96, %H = 4.00. Observed. %C = 75.01, %H = 4.03. FT-IR/ATR ν (cm^−1^): 3400–3000, 1650, 1590, 1227, and 1162 cm^−1^; TOF-MS: 13,459, C_840_H_534_O_117_ (Mw = 13,457); ^1^H-NMR (DMSO-*d*_6_) ppm: 9.02(s, 48H, -OH), 7.77–7.65 (m,177H, Ar), 7.20–7.09 (m, 126H, Ar), 7.05 (d, 48H, *J* = 8.8 Hz, Ar), 6.63 (m, 36H, Ar), 6.61 (m, 18H, Ar), 6.55 (m, 9H, Ar), 6.08 (t, 24H, *J* = 2.2 Hz, Ar), 5.93 (d, 48H, *J* = 2.2 Hz, Ar); ^13^C-NMR (DMSO-*d*_6_) ppm: 192.6 (C=O), 192.5 (C=O), 192.5 (C=O), 160.2 (Ar), 159.4 (Ar), 159.1 (Ar), 159.0 (Ar), 159.0 (Ar), 158.9 (Ar), 158.9 (Ar), 158.7 (Ar), 157.7 (Ar), 157.7 (Ar), 157.6 (Ar), 157.5 (Ar), 157.5 (Ar), 155.6 (Ar), 154.9 (Ar), 140.4 (Ar), 135.8 (Ar), 132.7 (Ar), 132.4 (Ar), 132.4 (Ar), 132.0 (Ar), 131.4 (Ar), 131.4 (Ar), 131.2 (Ar), 128.3 (Ar), 124.0 (Ar), 119.2 (Ar), 117.8 (Ar), 117.7 (Ar), 117.2 (Ar), 117.1 (Ar), 105.8 (Ar), 105.7 (Ar), 105.7 (Ar), 105.6 (Ar), 104.7 (Ar), 104.5 (Ar), 98.9 (Ar), 97.7 (Ar).

#### 3.2.3. Hyperbranched Poly(ether ether ketone) (**Hyper-OH**)

A mixture of **2** (1.62 g, 5 mmol), potassium carbonate (0.373 g, 2.7 mmol), toluene (10 mL) and *N*,*N*-dimethylacetamide (DMAc) (20 mL) was stirred in a flask at 120 °C. The temperature was then raised from 120 °C to 165 °C, to remove water formed during the reaction as an azeotrope with toluene. The reaction mixture was stirred at this temperature for 6 h. The polymerization proceeded homogeneously. After the reaction was completed, the mixture was cooled to room temperature and poured into 300 mL of methanol. The precipitated polymer was collected by filtration, washed thoroughly with water and methanol, and dried under vacuum. Yield 88%; white powder; Elemental Analysis: C_19_H_12_O_4_; Calculated %C = 74.99, %H = 3.97. Observed. %C = 74.52, %H = 3.64. FT-IR/ATR ν (cm^−1^): 3400–3000, and 1240 cm^−1^; ^1^H-NMR (DMSO-*d*_6_) ppm: 7.86–7.58 (m,4H, Ar), 7.30–7.00 (m, 4H, Ar), 6.72–6.64 (m, 0.51H, dendritic Ar), 6.36–6.31 (m, 1.03H, linear Ar), 6.31–6.24 (m, 0.51H, linear Ar), 6.08–6.04 (m, 0.32H, terminal Ar), 5.96–5.90 (m, 0.64H, terminal Ar), 5.93 (d, 48H, *J* = 2.2Hz, Ar).

#### 3.2.4. Hyperbranched Poly(ether ether ketone) (**Hyper-OMe**)

A mixture of **2** (1.62 g, 5 mmol), potassium carbonate (0.373 g, 2.7 mmol), toluene (10 mL) and DMAc (20 mL) was stirred in a flask at 120 °C. The temperature was then raised from 120 °C to 165 °C to remove water formed during the reaction as an azeotrope with toluene, and the reaction mixture was stirred at this temperature for 6 h. After the reaction mixture was cooled to room temperature, methyl sulfate (0.38 g, 3 mmol) and potassium carbonate (0.42 g, 3 mmol) were added. The mixture was stirred at 80 °C for another 6 h, cooled to room temperature, and poured into 300 mL of methanol. The precipitated polymer was collected by filtration, washed thoroughly with water and methanol, and dried under vacuum. Yield: 85%; white powder; Elemental Analysis: C_20_H_14_O_4_; Calculated %C = 75.46, %H = 4.43. Observed. %C = 75.05, %H = 4.23. FT-IR/ATR ν (cm^−1^): 2945, 1650 and 1240 cm^−1^; ^1^H-NMR (DMSO-*d*_6_) ppm: 7.86–7.58 (m,4H, Ar), 7.30–7.00 (m, 4H, Ar), 6.72–6.64 (m, 0.52H, dendritic Ar), 6.36-6.31 (m, 1.00H, linear Ar), 6.31–6.24 (m, 0.50H, linear Ar), 6.07–6.01 (m, 0.33H, terminal Ar), 6.00–5.93 (m, 0.65H, terminal Ar), 3.81–3.75 (s, 3H, -CH_3_).

#### 3.2.5. *1-(tert-Butyldimethylsilyloxy)-3,5-bis(4-fluorobenzoyl)benzene* (**4**)

A solution of *tert*-butyldimethylchlorosilane (4.97 g, 33 mol) in *N*,*N*-dimethylformamide (DMF) (20 mL) was added dropwise to a solution of **3** (10.15 g, 30 mmol) and imidazole (2.25 g, 33 mmol) in DMF (40 mL) at 0 °C. After stirring at 25 °C for 3 h, the precipitated imidazole salt was removed by filtration, and the solvent was evaporated. The residue was distilled under reduced pressure (glass tube oven) to pure 1-(*tert*-butyldimethylsilyloxy)-3,5-bis(4-fluorobenzoyl)benzene. Yield 72%; transparent oil; Bp: 240 °C (1.0 mmHg); Elemental Analysis: C_26_H_26_F_2_O_3_Si; Calculated %C = 69.00, %H = 5.79. Observed. %C = 68.72, %H = 5.61. FT-IR/ATR ν (cm^−1^): 3060, 2930, 1650, 1600, 1230, and 1150 cm^−1^; TOF-MS: 451, C_26_H_26_F_2_O_3_Si (Mw = 452.6); ^1^H-NMR (CDCl_3_) ppm: 7.88 (m,4H, Ar), 7.65 (t, 1H, *J* = 8.8 Hz, Ar), 7.54 (d, 2H, *J* = 8.8 Hz, Ar), 7.17 (m, 4H, Ar), 0.96 (s, 9H, -CH_3_), 0.09 (s, 6H, -CH_3_); ^13^C-NMR (CDCl_3_) ppm: 194.0 (C=O), 160.5 (d, *J* = 254 Hz) (Ar), 159.5 (Ar), 139.4 (Ar), 133.0 (d, *J* = 3 Hz) (Ar), 132.5 (d, *J* = 10 Hz) (Ar), 123.7 (Ar), 118.5 (Ar), 116.0 (d, *J* = 22 Hz) (Ar), 34.6(-CH_3_), 32.8(-CH_3_).

#### 3.2.6. Linear poly(ether ketone) Having Hydroxyl Groups in the Side Chains (**Linear-OH-BisA**)

A mixture of **4** (1.13 g, 2.5 mmol), bisphenol A (0.571 g, 2.5 mmol), potassium carbonate (0.345 g, 2.5 mmol), toluene (10 mL) and DMAc (20 mL) was stirred in a flask at 120 °C. The temperature was then raised from 120 °C to 165 °C, to remove water formed during the reaction as an azeotrope in toluene. The reaction mixture was stirred at this temperature for 6 h. The polymerization proceeded homogeneously. The mixture was cooled to room temperature, and poured into water (300 mL). The precipitated polymer was collected by filtration, and dried under vacuum. Yield: 95%; white powder; FT-IR/ATR ν (cm^−1^): 3060, 2930, 1650, 1600, 1230, and 1150 cm^−1^; ^1^H-NMR (DMSO-*d*_6_) ppm: 8.10–7.70 (m,3H, Ar), 7.50–7.30 (m, 4H, Ar), 7.30–6.96 (m, 12H, Ar), 3.4 (s, 6H,-CH_3_), 0.90 (s, 9H, -CH_3_), 0.15 (s, 6H, -CH_3_). Hydrochloric acid (5 M, 3.0 mL) was added to a solution of the obtained poly(ether ketone) (1.50 g) in DMF (25 mL), and stirred at 80 °C for 3 h. The reaction mixture was poured into methanol (250 mL). The precipitated polymer was collected by filtration and dried under vacuum. Yield 92 %; white powder; Td 360 °C; Elemental Analysis: C_35_H_26_O_5_; Calculated %C = 79.83, %H = 4.98. Observed %C = 79.40, %H = 4.98. FT-IR/ATR ν (cm^−1^): 3600–3200, 3060, 2930, 1650, 1600, 1230, and 1150; ^1^H-NMR (DMSO-*d*_6_) ppm: 8.20–7.70 (m, 4H, Ar and -OH), 7.50–7.30 (m, 4H, Ar), 7.30–6.90 (m, 12H, Ar), 3.35 (s, 6H, -CH_3_).

#### 3.2.7. Linear Poly(ether ketone) with Methoxy Groups in the Side Chains (**Linear-OMe-BisA**)

Compound **5** (0.846 g, 2.5 mmol), bisphenol A (0.571 g, 2.5 mmol), potassium carbonate (0.345 g, 2.5 mmol), toluene (10 mL) and DMAc (20 mL) were stirred in a flask at 120 °C. The temperature was then raised from 120 °C to 165 °C to remove water formed during the reaction as an azeotrope in toluene. The reaction mixture was stirred at this temperature for 6 h. The polymerization proceeded homogeneously. After the reaction was completed, the mixture was cooled to room temperature, and poured into water (300 mL). The precipitated polymer was collected by filtration, and dried under vacuum. Yield 93%; white powder; Td 370 °C; Elemental Analysis: C_36_H_28_O_5_; Calculated %C = 79.98, %H = 5.22. Observed %C = 79.65, %H = 5.05. FT-IR/ATR ν (cm^−1^): IR(liquid film): 3060, 2930, 1650, 1600, 1230, and 1150 cm^−1^; ^1^H-NMR (DMSO-*d*_6_) ppm: 8.10–7.70 (m, 3H, Ar), 7.50–7.30 (m, 4H, Ar), 7.30–6.96 (m, 12H, Ar), 3.93 (s, 3H, -OCH_3_), 3.35 (s, 6H, -CH_3_).

## 4. Conclusions

1-(*tert*-Butyldimethylsiloxy)-3,5-bis(4-fluorobenzoyl)benzene was polycondensed with bisphenols, followed by cleavage of the protective group to form linear poly(ether ketone)s, and the properties, such as solubilities, reduced viscosities and thermal properties, were compared with those of poly(ether ether ketone) dendrimer having hydroxyl chain ends and hyperbranched poly(ether ether ketone) having hydroxyl chain ends. A similar comparison was carried out with the corresponding methoxy group polymers. The solubilities of the dendrimers and the hyperbranched polymers were higher than the linear polymers. The reduced viscosities in DMAc of the hyperbranched polymers and the linear polymers increased with the concentrations, but those of the linear polymers increased more significantly. The order of increasing *T*_g_s was hyperbranched polymers > linear polymers > dendrimers, and the hydroxyl linear polymers crystallized thermally. The molecular size was shown to affect their properties.
